# Predicting postoperative systolic dysfunction in mitral regurgitation: CT vs. echocardiography

**DOI:** 10.3389/fcvm.2024.1297304

**Published:** 2024-02-23

**Authors:** Prajwal Reddy, Vidhu Anand, Prabhakar Rajiah, Nicholas B. Larson, Jared Bird, James M. Williams, Eric E. Williamson, Rick A. Nishimura, Juan A. Crestanello, Arman Arghami, Jeremy D. Collins, Alex Bratt

**Affiliations:** ^1^ Department of Cardiovascular Medicine, Mayo Clinic, Rochester, MN, United States; ^2^ Department of Radiology, Division of Cardiovascular Imaging, Mayo Clinic, Rochester, MN, United States; ^3^ Department of Quantitative Health Sciences, Mayo Clinic, Rochester, MN, United States; ^4^ Department of Cardiovascular Surgery, Mayo Clinic, Rochester, MN, United States

**Keywords:** computed tomography, mitral annuloplasty, echocardiography, ejection fraction, systolic dysfunction

## Abstract

**Introduction:**

Volume overload from mitral regurgitation can result in left ventricular systolic dysfunction. To prevent this, it is essential to operate before irreversible dysfunction occurs, but the optimal timing of intervention remains unclear. Current echocardiographic guidelines are based on 2D linear measurement thresholds only. We compared volumetric CT-based and 2D echocardiographic indices of LV size and function as predictors of post-operative systolic dysfunction following mitral repair.

**Methods:**

We retrospectively identified patients with primary mitral valve regurgitation who underwent repair between 2005 and 2021. Several indices of LV size and function measured on preoperative cardiac CT were compared with 2D echocardiography in predicting post-operative LV systolic dysfunction (LVEF_echo_ <50%). Area under the curve (AUC) was the primary metric of predictive performance.

**Results:**

A total of 243 patients were included (mean age 57 ± 12 years; 65 females). The most effective CT-based predictors of post-operative LV systolic dysfunction were ejection fraction [LVEF_CT_; AUC 0.84 (95% CI: 0.77–0.92)] and LV end systolic volume indexed to body surface area [LVESVi_CT_; AUC 0.88 (0.82–0.95)]. The best echocardiographic predictors were LVEF_echo_ [AUC 0.70 (0.58–0.82)] and LVESD_echo_ [AUC 0.79 (0.70–0.89)]. LVEF_CT_ was a significantly better predictor of post-operative LV systolic dysfunction than LVEF_echo_ (*p* = 0.02) and LVESVi_CT_ was a significantly better predictor than LVESD_echo_ (*p* = 0.03). Ejection fraction measured by CT demonstrated significantly greater reproducibility than echocardiography.

**Discussion:**

CT-based volumetric measurements may be superior to established 2D echocardiographic parameters for predicting LV systolic dysfunction following mitral valve repair. Validation with prospective study is warranted.

## 1 Introduction

Mitral regurgitation (MR) is the most frequently recognized valvular heart disease in the United States (1). Left untreated, chronic left ventricular (LV) volume overload from MR eventually leads to irreversible myocardial remodeling and contractile dysfunction (2). Early surgical repair has been shown to improve survival (3) though the optimal timing of operation is not well understood. Current guidelines from the American College of Cardiology and American Heart Association recommend mitral valve repair based on 2D echocardiographic thresholds of left ventricular ejection fraction (LVEF_echo_) and end systolic diameter (LVESD_echo_) as these have been shown to predict the occurrence of post-operative LV dysfunction (4, 5). Current thresholds are 60% for LVEF_echo_ and 40 mm for LVESD_echo_. By nature, 2D measurements contain only a small fraction of the information necessary to fully describe ventricular size and function and therefore likely fail to capture all the adverse myocardial remodeling that occurs in MR. Thus, we hypothesize that volumetric parameters are better predictors of outcome and could improve risk stratification in patients with MR.

Unlike echocardiography, Cardiac CT is inherently volumetric and insensitive to operator skill. Cardiac CT is already used to preoperatively evaluate coronary artery disease in appropriately selected patients undergoing mitral repair since it reduces the need for invasive coronary angiography (6). At our institution, pre-procedural cardiac CT is routinely performed with time-resolved retrospective ECG gating to allow for characterization of mitral leaflet structure and function and also to compensate for the high prevalence of arrhythmia in these patients (7–9). Time-resolved imaging also enables volumetric assessment of ventricular chamber size and function. This gives a more complete picture of LV status than 2D echocardiographic measurements and could clarify the optimal timing of intervention by improving prediction of irreversible LV remodeling. Motivated by this possibility, we sought to compare CT- and echocardiography-based measures of left ventricular size and function as predictors of LV systolic dysfunction following repair of primary MR.

## 2 Materials and methods

This research protocol was carried out under the supervision of our Institutional Review Board (IRB), which approved retrospective analysis of pre-existing datasets and waived the requirement for informed consent (IRB# 21-001262, deemed exempt 02/25/21). Patients who refused research participation were excluded from analysis according to state law.

### 2.1 Patient selection

**Figure 1:**
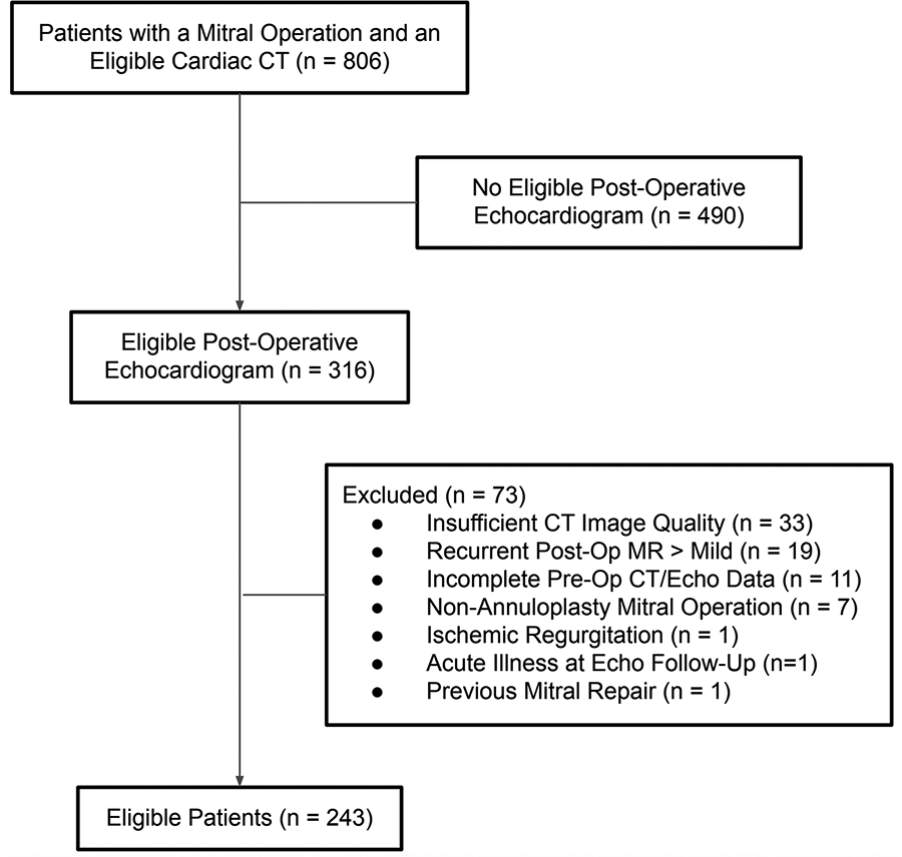
Flow chart showing patients included in the study. MR, mitral regurgitation.

Patient selection is summarized in Figure 1. Briefly, a medical record query was performed to identify patients for analysis. A patient was tentatively deemed eligible if her medical record contained a cardiac CT report issued between 2005 and 2022 that contained the words “mitral” and “prolapse”, along with a pre-operative diagnosis note that mentioned “mitral” (*n* = 806). Patients were excluded whose medical records did not contain at least one echocardiogram (transthoracic or transesophageal) performed between 6 and 36 months following the operation (*n* = 490). The charts of the remaining 316 patients were manually analyzed for additional exclusion criteria, including: insufficient CT image quality (*n* = 33; usually poor gating related to arrhythmia), incomplete pre-operative CT or echocardiographic data (*n* = 11), non-annuloplasty mitral operation (*n* = 7), ischemic etiology of MR (*n* = 1), acute illness at echo follow-up (*n* = 1), and previous mitral repair (*n* = 1). Additionally, 19 patients were excluded for recurrent post-operative MR of greater than mild severity since this could mask underlying systolic dysfunction. All cases of recurrent MR were independently reviewed by a level three trained echocardiographer blinded to outcome. The relatively high observed prevalence of recurrent MR (∼6%) was likely related to the criterion of a 6–36-month post-operative echocardiogram, which biases toward high-risk patients (e.g., those with symptoms, murmur, and residual regurgitation at hospital discharge). The total number of eligible patients after exclusion was 243.

### 2.2 CT imaging and analysis

Cardiac CT scans were performed according to an established protocol described elsewhere (6). Briefly, all examinations were performed using a dual-source, 64- or 96-detector row CT scanner (Siemens SOMATOM Force, Definition Flash, Definition; Forchheim, Germany). Patients were positioned supine with electrocardiogram (ECG) leads placed on the anterior chest wall. Scan parameters included variable kV (80–120) and mAs/rotation (150–400) as determined by patient size (using standard technique charts) to maintain a consistent quality reference mass. Pitch was also variable and was auto selected based on the patient's heart rate. All studies utilized intravenous iodinated contrast administration and were acquired in arterial phase. All studies employed retrospective ECG gating with tube current pulsing. An analysis of radiation dose for these exams has been previously described (6).

**Figure 2:**
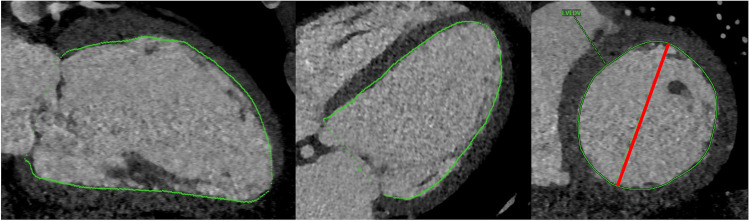
Depiction of chamber volume segmentation (green) and short axis diameter measurement (red).

Manual CT analysis was carried out by two raters blinded to patient outcomes, one of which a board-certified cardiothoracic radiologist with 2 years of post-fellowship experience (AB) and the other a fourth-year cardiology fellow undergoing subspecialty training in multimodality cardiovascular imaging (PRe). End-systolic volume (LVESV_CT_) and end-diastolic volume (LVEDV_CT_) were derived from CT scans using commercial semi-automated 3-D segmentation software (Visage, Richmond, Australia; Figure 2). LV short axis area was manually segmented at a few locations along the ventricle and the software interpolated between the manually segmented slices. After applying slight manual corrections to the interpolated segmentation maps, LV chamber volume was calculated by taking the integral of short axis area over the length of the ventricle. Chamber segmentation maps included trabeculation and papillary muscle as part of the chamber. LV ejection fraction (LVEF_CT_) was calculated in the standard fashion. The maximum LV short axis diameter was recorded in end-systole and end-diastole (LVEDD_CT_, LVESD_CT_, Figure 2).

### 2.3 Echocardiography image analysis and endpoint

Pre- and post-operative echocardiographic parameters were obtained from existing interpretive reports in the electronic medical record, including LVEF_echo_ (2D linear or biplane), 2D linear LV end diastolic diameter (LVEDD_echo_), and 2D linear LV end systolic diameter (LVESD_echo_). Echocardiographic volumes (LVEDV and LVESV) were not recorded because they were only available for a small minority of cases, even upon re-review of the original images. The endpoint of the study was post-operative left ventricular dysfunction. This was defined as a post-operative LVEF_echo_ of less than 50% measured on echocardiography performed between 6 and 36 months after the operation. If multiple post-operative echocardiograms were available during this time interval, we used LVEF_echo_ from the echocardiogram performed nearest to one year after the operation. All echocardiographic measurements were performed according to current American Society of Echcardiography guidelines (10).

### 2.4 Surgical repair

All patients underwent surgical repair of the mitral valve using a combination of leaflet resection, artificial chordae placement and flexible posterior annuloplasty band placement. Most operations were performed robotically (*n* = 188, 78%) (11), while 34 (14%) were performed via sternotomy and the remainder via port access. The robotic platform was introduced at our institution in 2008.

### 2.5 Reproducibility analysis

A random 50-patient cohort was identified for inter-rater reproducibility analysis. A single cardiothoracic radiologist with 13 years of post-fellowship experience (PRa) remeasured LVESV_CT_, LVEDV_CT_, and LVEF_CT_ for all patients in the reproducibility cohort. Another rater (PRe) remeasured preoperative LVEDD_echo_, LVESD_echo_, and LVEF_echo_ for all patients in the reproducibility cohort.

### 2.6 Statistical analysis

Diagnostic performance was assessed by means of receiver operating characteristic (ROC) curve analysis, with area under the curve (AUC) as the primary metric. Comparison of paired AUC estimates was carried out using the method of DeLong (12). Comparisons between groups were made using the Wilcoxon signed-rank test for paired data. Probability of post-operative systolic dysfunction over ranges of individual LV parameters was estimated univariably with logistic regression under a generalized additive model using flexible thin-plate splines. Lin's concordance correlation coefficient (CCC) was used for assessment of inter-rater reproducibility. To compare inter-rater reproducibility across modalities, we applied a bootstrapping approach to characterize the precision about the difference in CCC estimates between CT and echocardiography while taking into consideration the paired nature of the data by using patient as the resampling unit. The 95% confidence interval (CI) was calculated using the adjusted bootstrap percentile method based on *B* = 5,000 bootstrap samples. Inter-modality (i.e., CT vs. echocardiography) reproducibility was assessed using the method of Bland and Altman (13), which yielded a mean difference and limits of agreement (LoA; mean ± 1.96 standard deviation). Baseline variables were normalized to BSA (14) where appropriate. For all comparisons a two-sided *p*-value threshold of 0.05 was used to indicate statistical significance. Statistical analyses were performed with R v4.0.3 (15) as well as the Python packages NumPy (16), SciPy (17), and Statsmodels (18).

## 3 Results

### 3.1 Baseline characteristics

Baseline characteristics are shown in Table 1. Mean age was 57 ± 12 years and 65 (27%) patients were female. Atrial Fibrillation was present in 15 (6%) patients, diabetes in 3 (1%) patients, and only 4 (2%) patients had a diagnosis of congestive heart failure. Pulmonary hypertension, defined as right ventricular systolic pressure (RVSP) greater than 50 mmHg, was present in 17/215 (8%) of patients in whom RVSP was available on preoperative echocardiography. Mitral prolapse was present in all cases. In most patients, MR was severe or greater by preoperative echocardiography (*n* = 240, 99%) with the remainder of cases moderate or moderate-severe. Among patients with preoperative NYHA functional classification documented in the medical record (*n* = 160/243), the mean NYHA class was 2.0 with 142/160 patients class II or greater (i.e., symptomatic). The mean Society of Thoracic Surgeons score in the cohort was 0.6%.

**Table 1:** Baseline characteristics of the 243-patient cohort. Percentages are shown in parentheses.

Characteristic	Value
Age	57 ± 12
Mean STS score	0.60%
Number of females	65 (27)
Mitral regurgitation >=severe	240 (99)
Postoperative ejection fraction <50%	25 (10)
Atrial fibrillation	15 (6)
Myocardial infarction	1 (<1)
Congestive heart failure	4 (2)
Hypertension	60 (25)
Peripheral vascular disease	9 (4)
Cerebrovascular disease	0
Dementia	0
Chronic pulmonary disease	8 (3)
Peptic ulcer	1 (<1)
Diabetes	3 (1)
Renal disease	2 (1)
Liver disease	0
Rheumatologic disease	1 (<1)
Cancer	15 (6)

STS, society of thoracic surgeons.

### 3.2 Postoperative outcomes

There was no mortality in 30 days, nor did any patient require dialysis post-operatively. Median cardiopulmonary bypass time was 72 (IQR 59–87) minutes and cross clamp time was 50 (IQR 41–61) minutes. The most common concomitant procedure was closure of a patent foramen ovale or atrial septal defect (*n* = 35, 14%). Ten patients (4%) had concomitant coronary artery bypass, 15 (6%) had left atrial appendage ligation, 8 (3%) had the Cox maze procedure, and 6 (2%) had tricuspid annuloplasty. Postoperative atrial fibrillation occurred in 51 (21%) patients while only 15 (6%) had a history of atrial fibrillation preoperatively. On pre-discharge echocardiography, 228 patients (94%) had less than moderate residual MR and no patient had severe regurgitation. Median length of hospital stay was 3 (IQR 3–4) days.

### 3.3 Prediction of post-operative LV systolic dysfunction

**Figure 3:**
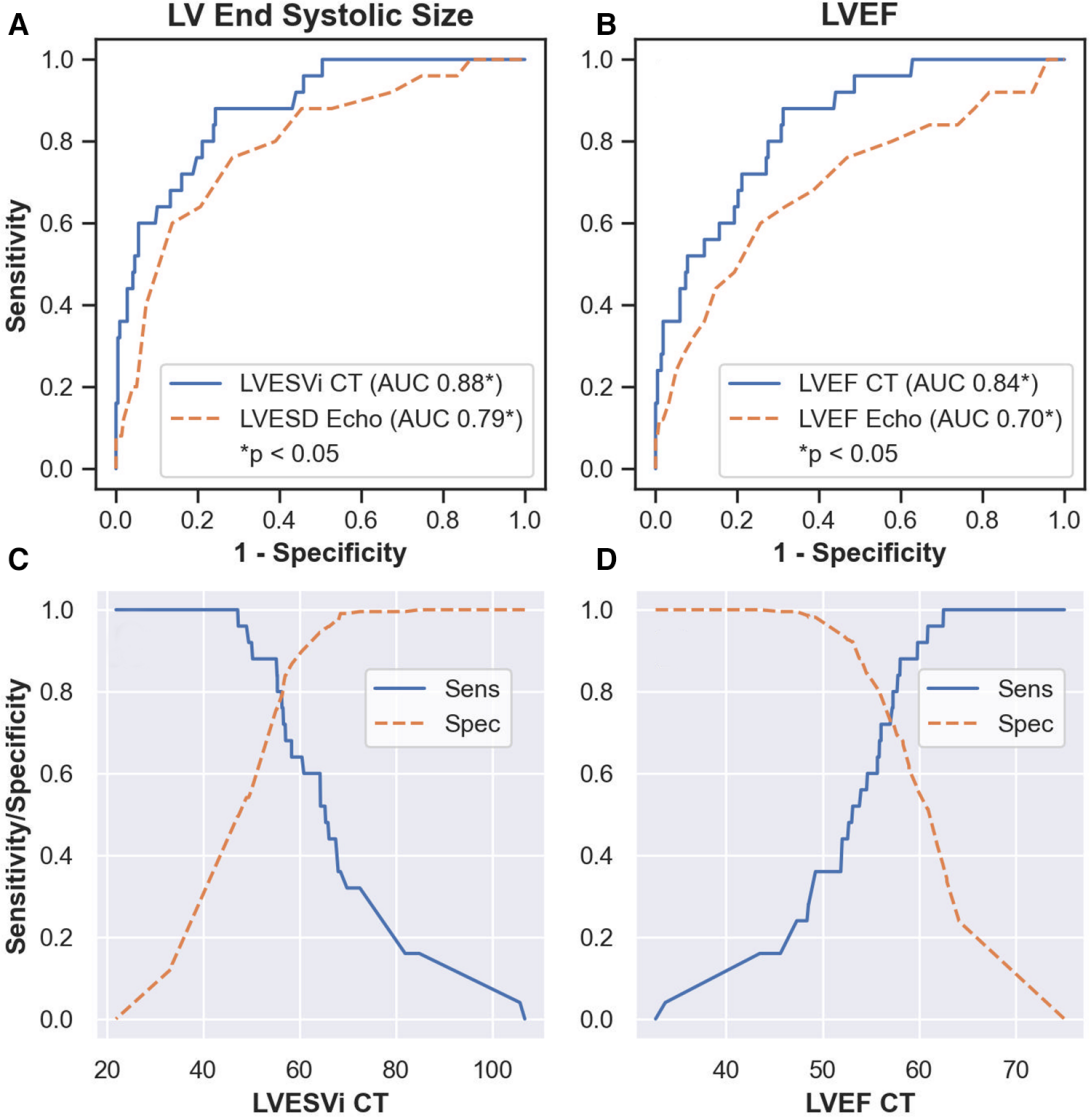
Results of receiver operating characteristic analysis. LVESVi_CT_ was a significantly better predictor of post-operative left ventricular dysfunction than LVESD_echo_ (*p* = 0.03, **A**). LVEF_CT_ was a significantly better predictor than LVEF_echo_ (*p* = 0.02, **B**). Sensitivity and specificity values over ranges of LVESVi_CT_ (**C**) and LVEF_CT_ (**D**) AUC, area under the receiver operating characteristic curve. LVEF_echo_, left ventricular ejection fraction on echocardiography. LVESD_echo_, left ventricular end systolic diameter on echocardiography. LVEF_CT_, left ventricular ejection fraction on CT. LVESVi_CT_, indexed left ventricular end systolic volume on CT.

**Figure 4:**
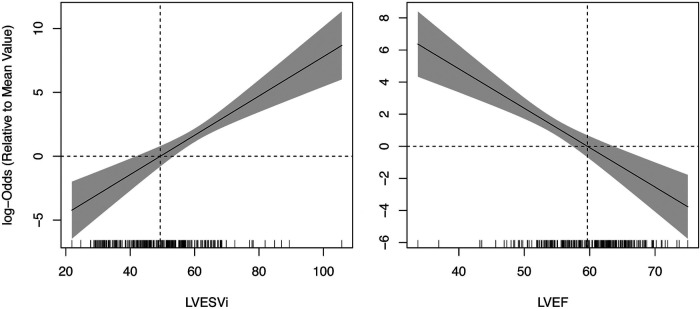
Logistic regression of post-operative systolic dysfunction vs. pre-operative CT-based LV measurements. The log-odds ratio of dysfunction (LVEF < 50%) increases linearly with LVESVi (left pane) and decreases with LVEF (right pane) across the entire range of observed values. Vertical dashed lines represent mean values of the study population. Horizontal dashed lines represent zero log odds relative to the sample mean. Hash marks along the *x*-axis represent individual data points. LV, left ventricle; LVEF, left ventricular ejection fraction; LVESVi, indexed left ventricular end systolic volume.

Twenty-five (10%) patients developed post-operative LV systolic dysfunction, defined as LVEF_echo_ less than 50%. Results from ROC analysis are shown in Table 2. BSA-indexed LVESV_CT_ (LVESVi_CT_) was a significantly better predictor of post-operative LV systolic dysfunction than LVESD_echo_ (AUC 0.88 vs. 0.79, *p* = 0.03; Figure 3). Non-indexed LVESV_CT_ was not a significantly better predictor than LVESD_echo_ (0.86 vs. 0.79, *p* = 0.08). Note that LVESD_echo_ was used for both comparisons since it was superior to indexed LVESD_echo_ in terms of AUC (0.79 vs. 0.68, *p* = 0.03). LVEF_CT_ was a significantly better predictor of post-operative LV systolic dysfunction than LVEF_echo_ (0.84 vs. 0.70, *p* = 0.02; Figure 3). Figure 4 shows the log-odds of post-operative systolic dysfunction over ranges of LVESVi_CT_ and LVEF_CT_, respectively. The log-odds of systolic dysfunction changes linearly with both parameters over both ranges of values, indicating that there was no threshold above or below which the risk of systolic dysfunction ceased to change as a function of these measurements.

**Table 2:** Results of ROC analysis.

Parameter	Original	BSA-indexed
LVEF (CT)	0.84 (0.77, 0.92)	–
LVEDV (CT)	0.74 (0.64, 0.84)	0.76 (0.67, 0.86)
LVESV (CT)	0.86 (0.79, 0.94)	0.88 (0.82, 0.95)
LVEDD (CT)	0.67 (0.56, 0.77)	0.59 (0.46, 0.72)
LVESD (CT)	0.75 (0.63, 0.87)	0.67 (0.55, 0.78)
LVEF (echo)	0.70 (0.58, 0.82)	–
LVEDD (echo)	0.69 (0.57, 0.80)	0.57 (0.44, 0.70)
LVESD (echo)	0.79 (0.70, 0.89)	0.68 (0.57, 0.80)

95% confidence intervals are shown in parentheses.

AUC, area under the ROC curve; CI, confidence interval; BSA, body surface area.

The LVESD_echo_ threshold value of 40 mm suggested by current guidelines (4, 5) yielded 60% sensitivity and 86% specificity in our cohort. At the same specificity level, LVESVi_CT_ gave a sensitivity of 68% (threshold value 58 ml/m^2^), identifying two additional patients with underlying systolic dysfunction (17 patients vs. 15). We propose 47 ml/m^2^ as a tentative LVESVi_CT_ threshold in our cohort as it provided 100% sensitivity with 50% specificity (Figure 3), while being over three standard deviations greater than the mean for healthy women and two standard deviations above the mean for healthy men (19). In our sample, this operative threshold would theoretically ensure that no patients develop post-operative LV dysfunction while exposing only 50% of patients to an earlier-than-ideal operation. Compare this to LVESD_echo_, which was only 13% specific at 100% sensitivity (cutoff 31 mm). This cutoff value is consistent with studies in patients with aortic regurgitation showing elevated risk at similar levels of indexed left ventricular end systolic volume (20–22).

The LVEF_echo_ threshold value of 60% suggested by current guidelines (4, 5) yielded 44% sensitivity and 85% specificity in our cohort. At the same specificity level, LVEF_CT_ gave a sensitivity of 56% (threshold LVEF_CT_ = 54%), identifying three additional patients with underlying systolic dysfunction (14 patients vs. 11). We propose 61% as a tentative LVEF_CT_ threshold in our cohort as this provides comparable specificity to the LVESVi_CT_ threshold described above (51%) while maintaining excellent sensitivity (96%, Figure 3). This cutoff value is consistent with findings in patients with aortic regurgitation showing elevated risk at a similar ejection fraction threshold (22).

Post-operative LV systolic dysfunction was associated with all baseline variables on univariate regression [*p* < 0.05; age, gender, LVEF_CT_, LVEDV_CT_, LVEDV_CT_ indexed to BSA (LVEDVi_CT_), LVESV_CT_, LVESVi_CT_, LVEDD_CT_, LVESD_CT_, BSA, LVEF_echo_, LVEDD_echo_, LVEDS_echo_]. However, the event rate was too limited to investigate multivariable models.

### 3.4 Inter-modality and inter-rater comparison

Correspondence between echocardiographic and CT measures of LV ejection fraction is shown in Supplementary Figure S2. The two modalities were significantly correlated (Pearson *r* = 0.4, *p* < 1e–11) with an estimated mean difference of −5% (CT minus echocardiography, *p* < 1e–26) and LoA ±11%.

Inter-rater reproducibility is summarized in Table 3. The 95% CI for the difference in CCC between EF measured by CT and echocardiography did not overlap zero (0.011, 0.522), indicating significantly greater reproducibility for LVEF_CT_ (0.76) than LVEF_echo_ (0.52).

**Table 3:** Results of inter-rater reproducibility analysis.

Modality	Measure	Lin's CCC	95% CI
CT	LVEF	0.76	0.63–0.85
CT	LVEDV	0.98	0.96–0.99
CT	LVESV	0.96	0.93–0.98
Echo	LVEF	0.52	0.32–0.68
Echo	LVEDV	0.82	0.71–0.90
Echo	LVESV	0.84	0.74–0.91

CCC, concordance correlation coefficient; CI, confidence interval.

## 4 Discussion

This is the first study to compare preoperative CT to echocardiography in predicting the outcome of surgical repair in patients with primary MR. The goal of surgical intervention in primary MR is to correct it before the onset of irreversible LV dysfunction (5). Early identification of patients at risk can be challenging because the chronic volume overload that accompanies MR artificially inflates LVEF, concealing underlying contractile dysfunction (23–26).

Current guidelines rely on preoperative 2D echocardiographic predictors of post-operative LV dysfunction (4, 23, 24, 27). However, technological advancements have enabled reproducible volumetric left ventricular measurements especially in patients who have an indication for multimodality imaging prior to surgical intervention. Preoperative cardiac CT not only allows for accurate assessment of coronary arteries, but retrospectively gated exams also allow for time-resolved volumetric assessment of the left ventricle. Our study suggests that CT-derived volumetric measurements better predict post-operative LV systolic dysfunction than established 2D echocardiographic parameters, which may clarify the optimal timing of repair. Importantly, most patients in our cohort began with normal or supranormal LVEF (Supplementary Figure S2), making this the exact group most likely to benefit from improved risk stratification. We also found that ejection fraction measured by CT demonstrated significantly greater reproducibility than echocardiography, highlighting another potential advantage of CT.

Use of CT for preoperative risk stratification may enable earlier intervention and thereby reduce the incidence of postoperative LV dysfunction. For example, since the LVEF_echo_ threshold of 60% suggested by current guidelines was only 44% sensitive in our cohort, 56% of patients who developed systolic dysfunction may have been incorrectly classified (14/25). With CT, we could have theoretically identified and intervened upon three additional patients at risk with similar specificity (only 11/25 misclassified). We note that these thresholds could be adjusted to reduce the number of misclassified patients further, at the cost of specificity.

We identify volumetric LV measurement thresholds (LVESVi_CT_ > 47 ml/m^2^, LVEF_CT _< 61%) that may provide a more favorable balance of sensitivity and specificity in our cohort. These align with prior studies, which identified similar risk thresholds in patients with aortic regurgitation (20–22). Our results show that cardiac CT is a promising avenue toward improving outcomes for patients with MR, though further validation is necessary in prospective randomized cohorts.

### 4.2 Study limitations

This study has several limitations. First, 2D transthoracic echocardiography was used to measure post-operative LV ejection fraction, which may be less accurate than CT, MRI, or 3D echocardiography (28). Future studies will assess the latter modalities as post-operative endpoints. Second, we did not evaluate preoperative 3D echocardiographic LV chamber measurements. This is because our goal was to compare with established guidelines (5), which are based on 2D measurements. Third, 11% of patients were excluded due to poor CT image quality, which could limit the benefit of CT. Given that CT exams in our study were not explicitly optimized for chamber volume measurement at the time of image acquisition, we feel that this could be mitigated with appropriate planning. Fourth, this study has all the inherent limitations associated with retrospective analysis of a relatively small sample size, including bias related to selection criteria. We plan to address this in future prospective studies.

Finally, CT exposes patients to ionizing radiation while echocardiography does not (29). The slight incremental risk incurred by radiation exposure is offset by the fact that preoperative cardiac CT reduces the need for invasive coronary angiography in patients undergoing mitral repair (6). One could argue that prospective gating would be a better choice for coronary evaluation since it delivers less radiation and maintains similar diagnostic accuracy (29). However, the additional radiation exposure of retrospective gating as compared with prospective gating is offset by several factors. First, retrospectively gated cardiac CT provides time-resolved information about mitral valve anatomy and function while prospectively gated CT does not. Second, retrospective gating is often already necessary for coronary evaluation in this patient population because of the relatively high prevalence of arrhythmia (7–9). Third, use of prospective gating does not allow dynamic assessment of LV volume and function, which improves prediction of post-operative LV dysfunction, as we show. If radiation remains a concern in certain settings, 3D echocardiography or cardiac MRI could be considered, especially given the value of the latter in assessing the severity of MR (30). This could be a subject for further study.

### 4.3 Conclusion

We provide evidence that CT-derived parameters may be more effective than standard of care echocardiographic evaluation of LV size and function in predicting post-operative left ventricular systolic dysfunction following mitral repair. With further validation, CT could inform clinical decision making by increasing diagnostic confidence and thereby enabling earlier intervention for patients at risk. After validation in larger prospective studies, incorporating volumetric CT-based measures of cardiac chamber size and function into routine preoperative evaluation may improve outcomes by reducing the incidence of post-operative LV systolic dysfunction.

## Data Availability

The data analyzed in this study is subject to the following licenses/restrictions: Mayo Clinic patient data. Requests to access these datasets should be directed to bratt.alexander@mayo.edu.

## Supplementary material

Supplemental Figure S1 Non-log odds ratio of post-operative systolic dysfunction versus CT-based LV measurements. The reference value for odds of post-operative systolic dysfunction (i.e., odds ratio = 1, horizontal dashed lines) was arbitrarily assigned to the sample mean for each measurement parameter (vertical dashed lines). Hash marks along the *x*-axis represent individual data points. The fact that these curves appear to have relatively flat segments as well as relatively vertical segments is an artifact of truncating the *x*-axes. As shown in Figure 4, consistent impacts on risk were observed across the spectrum of observed values. LV, left ventricle; LVEF, left ventricular ejection fraction; LVESVi, indexed left ventricular end systolic volume.

Supplemental Figure S2 Correspondence between echocardiographic and CT measures of left ventricular ejection fraction. The two modalities were significantly correlated (left plot) with mean difference −5% (CT minus echo) and limits of agreement ±11% (right plot). LVEF, left ventricular ejection fraction.
